# Supplementary figures and images for: Carbon fixation and rhodopsin systems in microbial mats from hypersaline lakes Brava and Tebenquiche, Salar de Atacama, Chile
Source: PLoS One. 2021 Feb 9;16(2):e0246656. doi: 10.1371/journal.pone.0246656 (PMC7872239; doi:10.1371/journal.pone.0246656)

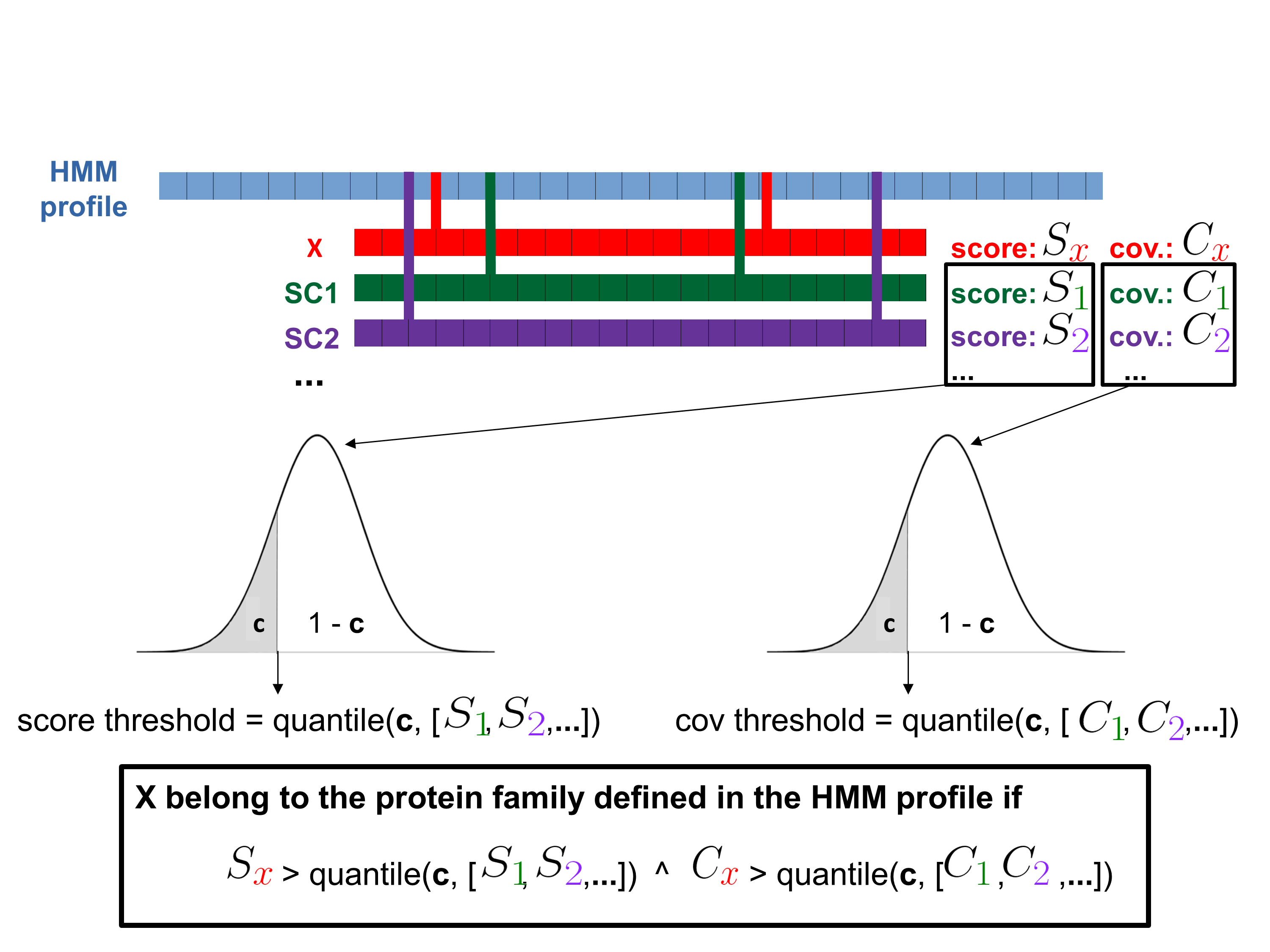

Supplement: S1 Fig — X being the fragment to be classified, it will belong to a certain protein family if its score and coverage are greater than the c quantile of the score (S) and coverage (C) of the set of positive fragments, which have the length of X and the extremes of their alignment contain those of X, or are contained by those of X. (TIF) [file pone.0246656.s005.tif]

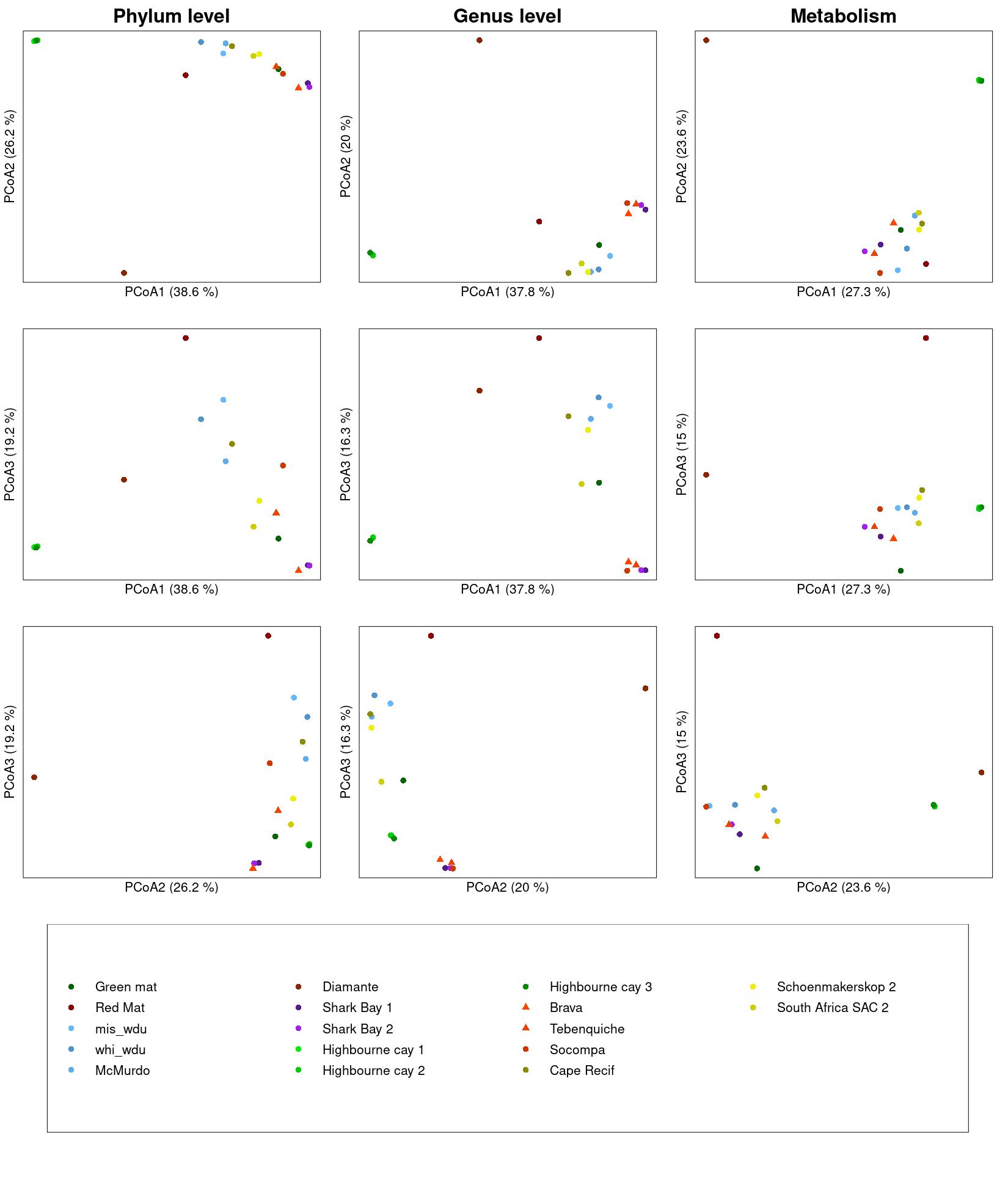

Supplement: S2 Fig — (TIF) [file pone.0246656.s006.tif]

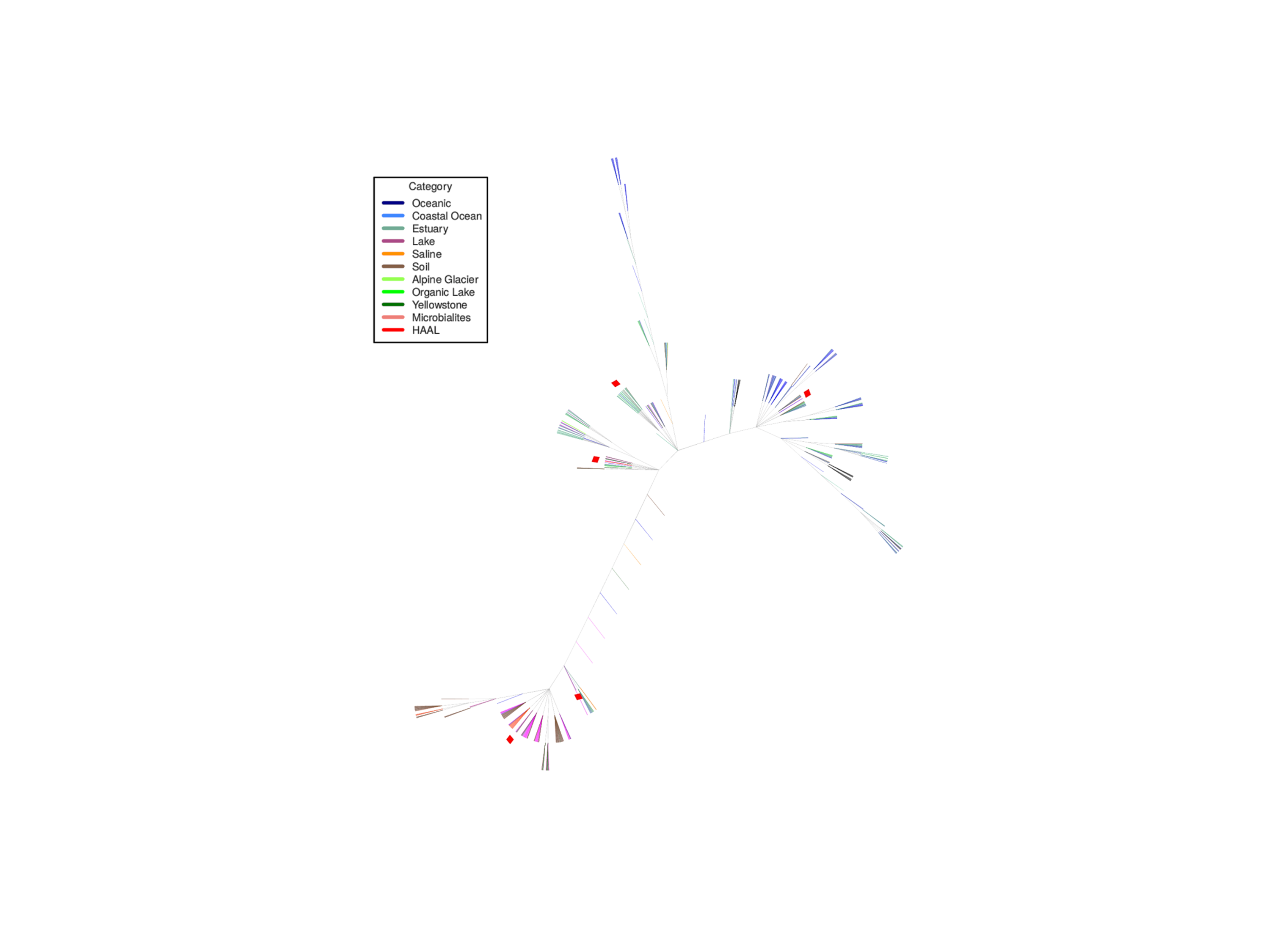

Supplement: S3 Fig — (TIF) [file pone.0246656.s007.tif]

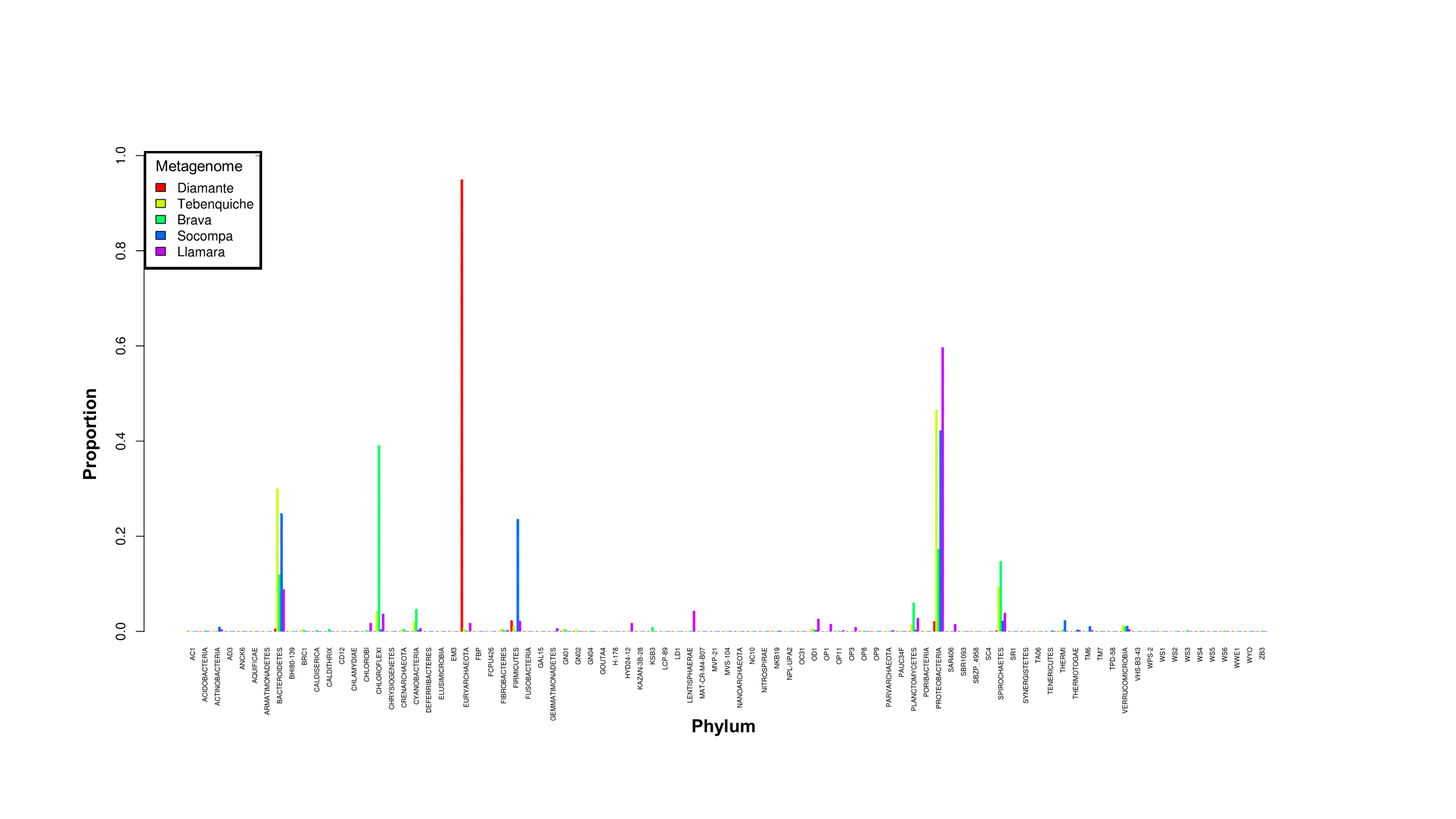

Supplement: S4 Fig — (TIF) [file pone.0246656.s008.tif]
