# Supplementary material for: Carbon fixation and rhodopsin systems in microbial mats from hypersaline lakes Brava and Tebenquiche, Salar de Atacama, Chile
Source: PLoS One. 2021 Feb 9;16(2):e0246656. doi: 10.1371/journal.pone.0246656 (PMC7872239; doi:10.1371/journal.pone.0246656)
Supplement: S1 File — (DOCX) [file pone.0246656.s009.docx]

Supplementary Methods

*Rhodopsin/recA/rad51 detection on metagenomic reads*

HMMer is a fast and accurate tool to classify sequences, but its application to short fragments derived from metagenomic sequences represents a challenge. HMMER results include bit-score (score), e-value and alignment region of the evaluated sequence to the selected profile. The proportion of aligned aminoacids will be here called “coverage”. The interaction of these three parameters and fragment length was analyzed with a set of fragments of rhodopsin sequences (the “positive dataset”, see below). Even though shorter fragments had greater coverage, they also had lowest scores and highest e-values (Fig. SM1 a, b, c). These parameters were also influenced by the alignment position to the HMM model (Fig. SM1 d).


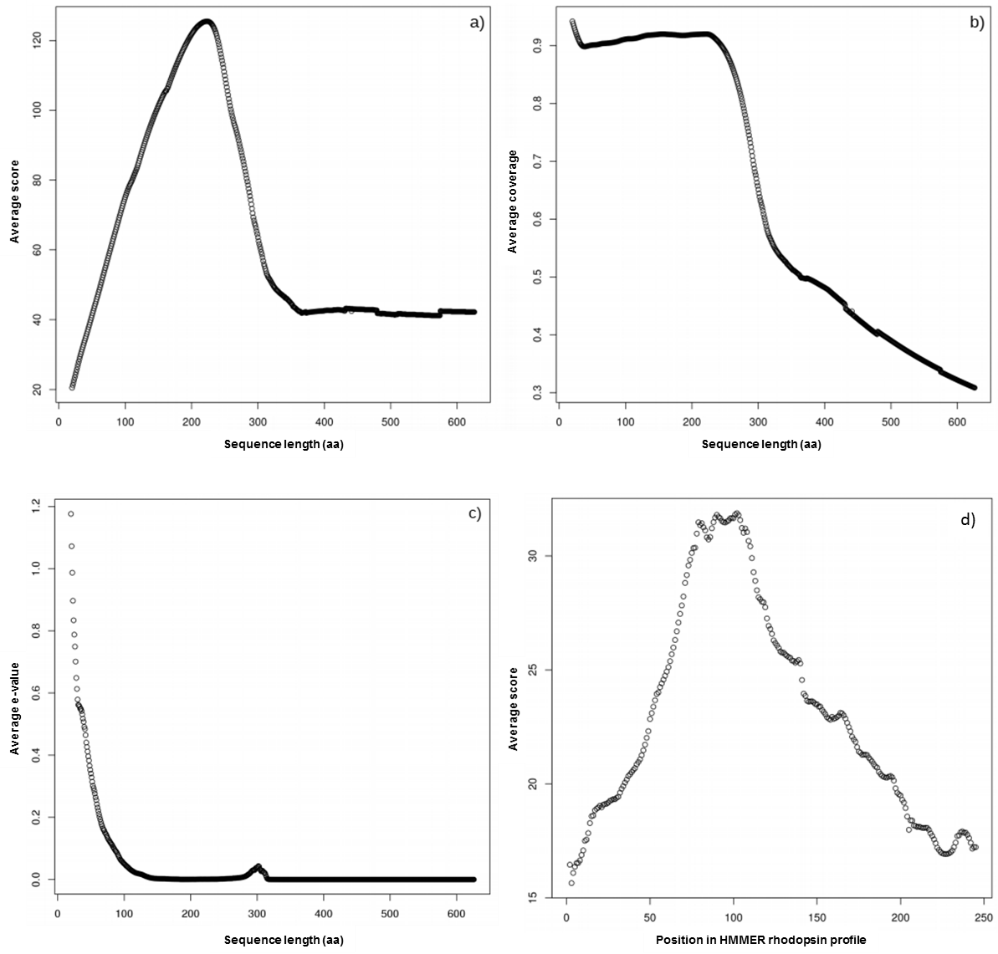


**Figure SM1.** HMMER results for rhodopsin fragments (positive dataset). Average score (a), coverage (b), and e-value (c) are drawn versus fragment sequence length. Average scores varied with the position on the MicRhode HMM profile.

Thus, the classification method was conceived as: X being the fragment to be classified, it will be considered rhodopsin if its score and coverage are greater than the C quantile of the score and coverage of the set of positive fragments, which have the length of X and the extremes of their alignment contain those of X, or are contained by those of X. The value of C was fixed by analyzing precision and sensitivity for a range of values.

To analyze the precision and sensitivity of the prediction two datasets were generated for each protein family analized. A set of positive sequences was obtained using the HMMER web service in Uniprot, with the selected models and a maximum e-value of 1E-05. For recombinases, additional filters were considered: only hits from Bacteria (tax id: 2) for RecA, and from Eucariota (tax id: 2759) and Archaea (tax id: 2157) for Rad51. Sequences not annotated as rhodopsins or recombinases were discarded, and also those used to build the HMM profiles. With these parameters, a set of 1908 rhodopsin sequences was obtained, and from the many RecA and Rad51 sequences, 500 from each were randomly chosen, with length below 600 aa. All these sequences were fragmented with all possible fragment lengths, starting at 20 aa, and the repeated fragments were discarded, generating 17 million, 18 millon, and 22 millon fragments for rhodopsin, RecA and Rad51 positive datasets. For the negative set, sequences were retrieved with HMMER from Uniref50, with e-values between 10-3 and 103. Thus, these sequences have some similarity with the sequences of interest. The sequences annotated as members of the families of interest were discarded, and 100 sequences were randomly selected. All possible fragments with lengths starting at 20 aa were obtained, resulting in 2.3 millon of negative fragments for rhodopsin, 2.9 millon for RecA, and 3.3 millon for Rad51.

From these datasets, all fragments retrieved from a random positive sequence were classified along all fragments from a negative sequence. This was repeated 100 times with the same positive sequence and a different negative sequence. The remaining positive sequences were used to calculate scores and coverages and determine C quantiles. Precision was calculated as

Precision = (TP / P) / [(TP / P) + (FP / N)],

where TP are true positives or fragments that belonged to the family and were correctly classified, FP are false positives or fragments that did not belong to the family and were incorrectly classified, P is the total amount of evaluated fragments belonging to the family and N is the total amount of fragments not belonging to the family. Sensitivity was calculated as

Sensitivity = VP / P

Precision and sensitivity were calculated considering different C values (Fig. SM2). These are global values, variation with fragment length was also considered (Fig. SM3). Precision was reduced with shorter fragments. Considering global precision (Fig. SM2) above 0.9 and sensitivity ~0.86 for all families, C value were selected as 0.04 and 0.05 for rhodopsins and recombinases, respectively.


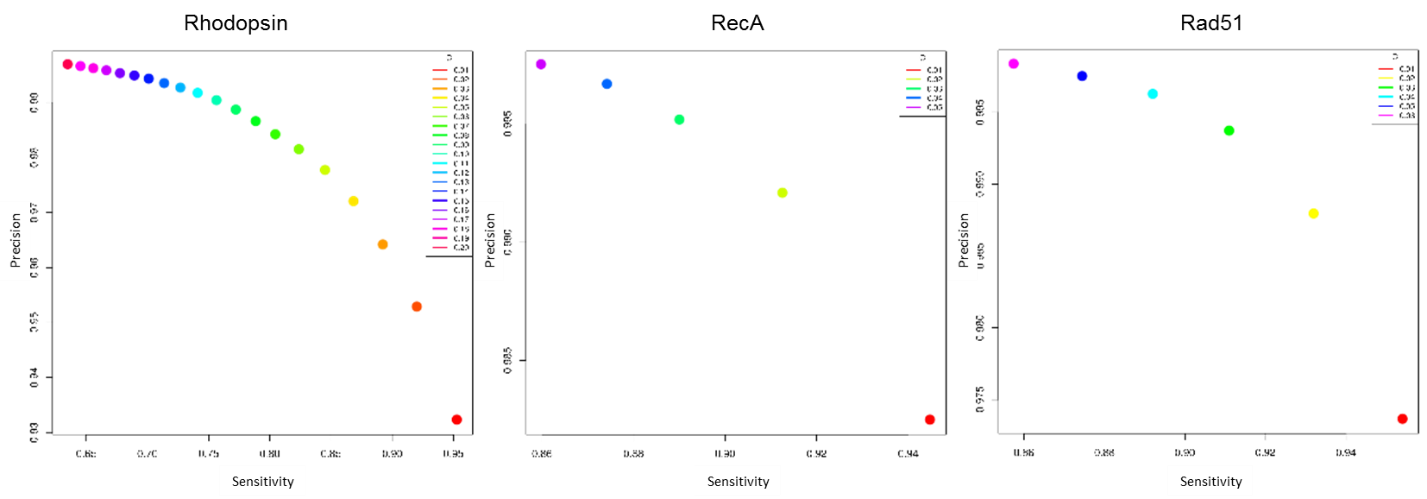


**Figure SM2.** Precision vs. Sensitivity for each family


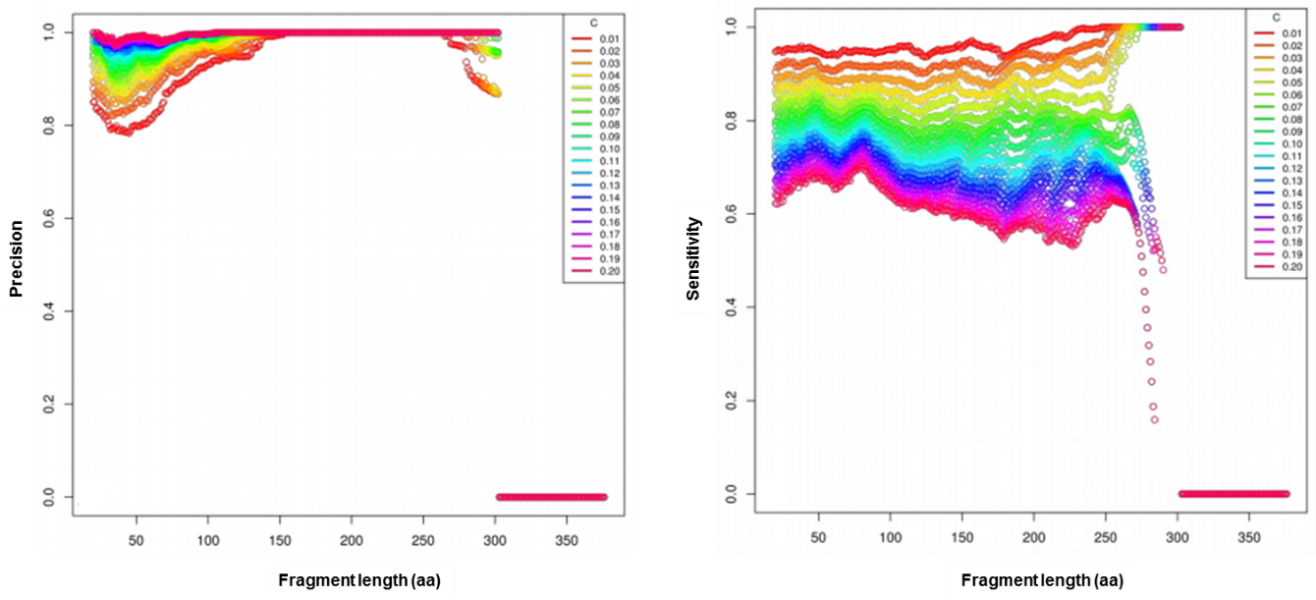


**Figure SM3.** Precision and Sensitivity for different fragment lengths in the rhodopsin family. No positive fragments larger than 300 aa, thus values deviate beyond 300.
