# Supplementary material for: Carbon fixation and rhodopsin systems in microbial mats from hypersaline lakes Brava and Tebenquiche, Salar de Atacama, Chile
Source: PLoS One. 2021 Feb 9;16(2):e0246656. doi: 10.1371/journal.pone.0246656 (PMC7872239; doi:10.1371/journal.pone.0246656)
Supplement: Attachment — Submitted filename: Response to reviewer.docx [file pone.0246656.s010.docx]

**Response to reviewer.**

Journal Requirements:

*1. Please ensure that your manuscript meets PLOS ONE's style requirements, including those for file naming.*

This has been addressed according to the requirements.

*2. We note that you have stated that you will provide repository information for your data at acceptance. Should your manuscript be accepted for publication, we will hold it until you provide the relevant accession numbers or DOIs necessary to access your data. If you wish to make changes to your Data Availability statement, please describe these changes in your cover letter and we will update your Data Availability statement to reflect the information you provide.*

All data is now publicly available. This was added to the cover letter.

*3. We note that you have included the phrase “data not shown” in your manuscript. Unfortunately, this does not meet our data sharing requirements. PLOS does not permit references to inaccessible data. We require that authors provide all relevant data within the paper, Supporting Information files, or in an acceptable, public repository. Please add a citation to support this phrase or upload the data that corresponds with these findings to a stable repository (such as Figshare or Dryad) and provide and URLs, DOIs, or accession numbers that may be used to access these data. Or, if the data are not a core part of the research being presented in your study, we ask that you remove the phrase that refers to these data.*

The phrase was removed and supplementary data (S2 table) was added.

*4. (October 2020) Thank you for including your ethics statement on the online submission form:
"At the moment of sampling the locations were freely accessible, with no protected areas from Chilean government, and no specific permissions were required for these activities."*

*To help ensure that the wording of your manuscript is suitable for publication, would you please also add this statement at the beginning of the Methods section of your manuscript file.*

The sentence was added as requested.

Reviewer comments on italics and our answers below. We appreciate the reviewer suggestions as they improved the manuscript, allowing us to correct several flaws.

*Overall recommendations:
1) All Figures – recommend to increase the resolution of all figures as they were extremely blurry and hard to see when zooming in. At 100% zoom, quite a few figures (e.g. figure 5), the text was very hard to read.*

Some figures were changed and resolution improved.

*2) Addition of a conclusion paragraph - there was no final conclusion statement – the manuscript abruptly ends (line 422), prompting me to search if I was missing a page. Recommend to add a conclusion paragraph that states the research question, results found supporting the study, and overall conclusions from the study.*

A conclusions section has been added

*3) Addition of minimal arsenic data - this manuscript mentions that these mats grow under high levels of ‘toxic metals’ and metaloids and the authors continuously mention arsenic in multiple places (e.g. introduction, line 52, 378-379; however, the study is primarily focused on carbon fixation and rhodopsin. Due to the fact that this is mentioned often, suggest putting in some minimal arsenic data (annotated arsenic genes, etc) to show that arsenic may indeed be tied to altitude – such as the same authors suggested in their 2017 study of arsenic metabolism in the HAAL system of Socompa.*

Arsenic is a major subject for the HAAL locations, and a comparative study including the data from this work is being carried out. A preprint is available and has been cited in the text (Line 86)

*4) Beta Diversity metrics – recommend to add an analysis of all sites together; explanation below.*

The analysis suggested by the reviewer was performed and added to the manuscript.

*5) Rhodopsin abundance per site – recommend to include an RHO abundance/site to corroborate if RHO is truly in higher abundances in HAAL environments. This result has the potential to positively influence the hypothesis of the study.*

Rhodopsin abundance was actually calculated and shown in figure 6. The result was poorly presented in the original manuscript and the figure has been changed in the revised version.

*Other specific comments to follow.
Abstract
Line 22: Please define WGS since it’s appearing for the first time in this manuscript.
Introduction*

Fixed.

*The introduction was very clear. It included a lot of background information and the purpose of the study was clear
– here are some recommendations for all sections of the manuscript:
Line 46: What is the altitude of the study sites? You mention this in the discussion, but adding altitude here would also be beneficial, especially since altitude is being mentioned.*

Data added.

*Lines 52-56: 1) Characterizes HAAL and is comparing this environment as similar to where the samples were collected. Suggest adding in at least another environment that is ‘extreme’ for comparison purposes, to avoid such a dichotomous comparison, such as the environment of Shark Bay, which is similar due to hyper salinity, etc and it is also used for comparison purposes in this study.*

Actually, the intention was to include the study sites within the broad HAAL category.

*2) Also, what is the altitude for the Puna Andrean region (line 53)?*

Data added.

*Line 58: characterize taxonomically to: taxonomically characterize*

Fixed.

*Materials and Methods
Line 105: Samples were pooled prior to sequencing – how many rounds of DNA extraction were conducted with the Power Biofilm Kit? Was 1 enough? (microbial mats are known to be rich in EPS).*

Added to text: “One round of extraction and pooling allowed obtaining enough DNA (pooled samples included DNA from three extractions, one from each triplicate core).”

*Line 116: What was the percent retained after paired end reads were merged?*

This was performed by the sequence provider and we do not have that data. Still, around 90% of the reads could be mapped to the assembly.

*Line 118: There is no coverage information given – suggest to include some coverage statistics (e.g. coverage/contig, average coverage, etc).*

Some data was added to S1 Table.

*Lines 122-125: 1) What alpha diversity metrics were used to assess the data? Line 235 states that alpha diversity had an observed value of 572 – what metric was used?*

This metric was provided by MG-RAST server, and it’s based on protein taxonomic annotations at the species level. A short paragraph was added to Materials and methods (see lines 131-135), describing the metric used.

*2) How was processing and filtering of data conducted (e.g. low abundance)?*

There was no processing other than performed by the MG-RAST server. Most analysis involve identifying specific features. Taxonomic analysis was rather superficial, only to present a broad view of the diversity, and in the figure 2 low abundance data (<1%) was grouped as "Minor phyla"

*Line 152 – 153: States – “Brava and Tebenquiche, and preprocess was required including adaptor trimming and quality filtering with Trimmomatic” Recommend changing to: …. and preprocessing, which included adaptor trimming and quality filtering ….*

Fixed.

*Line 169: In Supplemental Fig. 1: Suggest changing ‘X belong’ to ‘X belongs’.*

Fixed.

*Line 193: Please cite R. Run the command as is: citation() in R to show how to cite R.*

Fixed.

*Line 205-211: Calculating RHO abundance – was this calculation generated in house? Or was this borrowed from another publication? Your cite of [53] didn’t seem to include this RHO abundance ratio. If this ratio is noted elsewhere in the literature, please cite where it came from.*

Two citations added:

Sharma AK, Zhaxybayeva O, Papke RT, Doolittle WF. Actinorhodopsins: Proteorhodopsin-like gene sequences found predominantly in non-marine environments. Environ Microbiol. 2008;10: 1039–1056. doi:10.1111/j.1462-2920.2007.01525.x

Kwon S-K, Kim BK, Song JY, Kwak M-J, Lee CH, Yoon J-H, et al. Genomic makeup of the marine flavobacterium Nonlabens (Donghaeana) dokdonensis and identification of a novel class of rhodopsins. Genome Biol Evol. 2013;5: 187–99. doi:10.1093/gbe/evs134

*Results
For the ‘Taxonomic Diversity’ section, this section would be enhanced greatly if all metagenomes collected (Brava,Tebenquiche, Cuatro Cienegas, Diamante, Shark Bay Smooth and Pustular mats, HBC thrombolites, Socompa, Cape Recite, Schoenmakerskop, S. Africa, Markham Ice Shelf, Ward Hunt Ice Shelf, and McMurdo) were visually compared under a beta diversity metric (e.g. PCA, NMDS, PCoA, etc. with appropriate statistical testing) so that the following can be extrapolated: 1) percent variance and; 2) a visual representation of the ‘groupings’. This may be very informative to see the clustering patterns – if high altitude clusters together with Shark Bay mats or if there is a gradient of clustering on a metric. This might give clues as to the statements made in lines 283-284 about how the Wood-Ljungdahl were only observed in HAAL mats and in Shark Bay, at least in a taxonomic context.*

The suggested analysis was performed, see Metagenomic comparison sections at Materials and methods, and the Taxonomic and functional diversity section at Results.

*Line 223-224: Mentions that Kaiju assigned 45% and 51% of the reads. Suggest that MG-RAST assignment is also placed.*

Fixed*.*

*Line 243: Please remove the word ‘transcript’ in TPM. Unless this was an RNA-Seq analysis. Suggest replacing with reads per million or counts per million.*

Fixed*.*

*Line 251: Please define BM and TM, since it’s the first time appearing.*

Fixed*.*

*Line 269: What is considered ‘poor number of hits’?*

This meant a normalized abundance below 1 cpm, which would be barely visible on Fig 3B. The explanation was added to the text.

*Line 277: suggest changing to: finally, 4-hydroxybutyryl-CoA dehydratase (K14534), the key enzyme*
Fixed.

*Line 284: A note: Shark Bay mats are known to have this pathway via previous WGS in publications.*

Yes, the following reference was added:

Wong HL, White RA, Visscher PT, Charlesworth JC, Vázquez-Campos X, Burns BP. Disentangling the drivers of functional complexity at the metagenomic level in Shark Bay microbial mat microbiomes. ISME J. 2018;12: 2619–2639. doi:10.1038/s41396-018-0208-8

*Line 284: For Figure 4: Suggest delineating the classifications of each mat in the arbitrary grouping that is mentioned in line 148-149 or via environmental parameters (e.g. hypersaline, high altitude, etc.) to guide the reader as to what environment these mats are from.*

This was briefly mentioned in the text and added to Figure 4.

*Line 294: Suggest changing to: 182 and 253 coding sequences were found for rhodopsins in the metagenomic assemblies.*
Fixed.

*Line 300: ‘proteins; are mentioned – suggest indicating what proteins this refers to.*

Changed “proteins” to “sequences”*.*

*Line 324: Figure 6 is unclear and confusing. With increasing metagenomes, it is assumed Rhodopsin abundance will also increase.*

The figure presented all analyzed metagenomes sorted by abundance. “Increasing metagenomes” did not mean the sum of the abundances. We acknowledge that it was confusing and the figure was changed.

*This section would be greatly enhanced if data is also reported similarly as in Figure 4. For each of the locations in Figure 4, report what the RHO abundances are (as the Y axis). This may help to corroborate the thesis of this paper - if RHO is truly in higher abundances in HAAL environments (and perhaps even Shark Bay), where cyanobacterial abundances are low or lower than other microbial mat ecosystems. This result has the potential to have a strong influence on this area of study*

The abundance analysis involved hundreds of metagenomes, and thus is poorly presented in a bar graph as in Figure 4. The original Figure 6 was actually a bar graph. Thus, metagenomes were arbitrarily grouped (as already described in text) and a boxplot is presented, summarizing abundances observed for the different groups.
